# Effect of fabrication technique on surface roughness and color stability of novel resin occlusal veneers after thermomechanical aging: an in-vitro study

**DOI:** 10.1186/s12903-026-08536-8

**Published:** 2026-05-18

**Authors:** Ahmed Rizk Ali Mostafa, Badawy Abo El Mahasen Badawy, Mervat Mourad Roushdy

**Affiliations:** Fixed Prosthodontic Department, Faculty of Dentistry, Cairo University, 11 El-Saraya St, Manial, Cairo, 11553 Egypt

**Keywords:** Occlusal veneer, CAD/CAM, 3D printing, Resin-matrix material, Surface roughness, Color stability, Thermomechanical aging

## Abstract

**Background:**

Resin-based occlusal veneers are increasingly used within minimally invasive restorative dentistry. However, evidence remains limited regarding whether fabrication technique (subtractive milling versus additive 3D printing) influences clinically relevant surface characteristics particularly surface roughness and color stability after thermomechanical aging.

**Methods:**

Eighteen resin occlusal veneers were fabricated from the same standardized digital design (STL) using NanoKsa G-Plus material and divided into two groups (*n* = 9): milled CAD/CAM and 3D-printed. Measurement of surface roughness (Ra) was performed utilizing a non-contact profilometer at baseline and after thermomechanical aging while Color change (ΔE) was assessed with a spectrophotometer (Vita Easyshade) sequentially: after coffee immersion (1 week) and then repeated again after thermomechanical aging. Thermomechanical aging combined thermocycling (5000 cycles) and chewing simulation (75,000 cycles) under standardized loading. Intergroup comparisons were performed using independent t-tests, with level of significance determined at *p* ≤ 0.05 and 1.0 < ΔE*ab threshold range < 3.3 commonly reported clinically acceptable thresholds.

**Results:**

At baseline, the 3D-printed group showed significantly greater roughness of surface than the milled group (Ra: 0.27 ± 0.007 vs. 0.24 ± 0.02; *p* = 0.05). After thermomechanical aging, no significant difference was detected between groups (Ra: 0.26 ± 0.021 vs. 0.27 ± 0.016; *p* = 0.50). For color change, the 3D-printed group demonstrated significantly greater ΔE than the milled group across all intervals, including baseline-to-post-staining (34.33 ± 6.14 vs. 13.14 ± 2.31; `*p* = 0.001) and post-staining-to-post-aging (18.78 ± 3.85 vs. 2.66 ± 1.89; `*p* < 0.001`).

**Conclusions:**

Thermomechanical aging reduced differences in surface roughness between milled and 3D-printed resin occlusal veneers; however, 3D-printed specimens exhibited substantially higher discoloration after staining and aging. This may indicate that, despite comparable post-aging roughness, color stability may limit the clinical use of 3D-printed in situations where esthetics are critical indicating that their recorded stainability values exceeded commonly reported clinical acceptable thresholds.

**Supplementary Information:**

The online version contains supplementary material available at 10.1186/s12903-026-08536-8.

## Introduction

Minimally invasive restorative dentistry has increasingly shifted toward adhesive, defect-oriented strategies that preserve sound tooth structure while restoring function and esthetics. Within this framework, occlusal veneers (thin overlays/onlays) represent a conservative option for managing occlusal wear or morphology correction, provided that preparation design and bonding are optimized to support fatigue resistance and favorable stress distribution [1].

Undoubtedly there are various preparation designs in the literature but the most recent researches reached the fact that by using the partial coverage in the posterior teeth significantly minimize the loss of sound tooth structure up to 45% compared to the full coverage [2]. 

Occlusal veneers are indicated to be used as a reliable solution in moderate and severe compromised teeth while different materials share in their fabrication like glass ceramics (Emax), poly crystalline ceramics (Zirconia), resin ceramics and resin-based composites [3]. 

With the expansion of digital workflows, indirect restorations can be produced through subtractive manufacturing (CAD/CAM milling) or additive manufacturing (3D printing). In milling techniques, pre-polymerized solid blanks are situated in a milling unit where computer-controlled cutting tools configure the shape of prosthesis. On the other side, in the additive techniques, the 3D parts are constructed layer by layer till the final desired shape. Each layer is positioned accurately according to the digital design of the restoration, and the layers are united together subsequently to create the final restoration [4]. 

Previous studies indicate that the additively manufactured resin restorations can offer practical advantages (material efficiency, reduced waste, and potentially lower cost), yet their performance remains strongly dependent on printer technology, polymerization quality, and post-processing protocols [4]. One of the remarkable differences between milling and 3d printing approach is the meticulous post-processing step that is mandatory in 3D-printing, on the contrary this step is not needed in milling approach. Post-processing methods are essential in polymer-based 3D printing to boost the general mechanical properties and final outcome of printed objects [5]. 

Systematic evidence comparing printed to milled resin restorations highlights that, despite promising feasibility, stain susceptibility and surface-related outcomes remain key limitations requiring further clarification [5]. 

Nanoksa G-Plus is a biocompatible high-performance polymer reinforced with nano-carbon and nano-zirconia, and it is indicated to design a long-lasting restoration on dental implants. Nanoksa G-Plus is accessible in two forms either resin or disc. the resin form is indicated for veneers, inlays, onlays, and single crowns whereas the disc may undergo water sorption nearly 0.1% after 24 h of immersion in water at 23C^0^ reported by the manufacturer. Thanks to its purely crystalline nature Nanoksa G-Plus doesn’t dissolve in common solvents at normal rate temperature [6]. 

Nanoksa G-Plus is made up of a remarkable constituent of polymers that has positive impact on wear resistance, special notable strength characteristics, perfect biocompatibility, devoid of any monomer in their composition, low weight, higher mechanical properties, extended durability, and efficient shock absorption. This material poses a high aesthetic appearance and transparency that resemble the color of natural teeth compared to PEEK material which is considered one of its main drawbacks and Nanoksa also yields exceptional abrasion resistance and color stability [6]. 

Among the clinically relevant surface-related properties, surface roughness is critical because it affects plaque accumulation, gingival inflammation risk, and long-term restoration maintenance. A surface roughness threshold refers to an essential value of surface texture beyond which a material’s performance or biological interaction significantly changes. Various studies and researches have reported that the ideal threshold clinical value for surface roughness is 0.2 μm (Ra). Below this level, surface roughness does not considerably either induce plaque or biofilm formation, emphasizing the importance of keeping restorative surfaces as smooth as possible through fabrication accuracy and finishing/polishing protocols [7]. 

In parallel, color stability and stainability are essential determinants of patient satisfaction for resin-based restorations. Color change may originate from intrinsic factors (water sorption, resin matrix degradation, filler–matrix interface breakdown) and extrinsic factors (dietary chromogens such as coffee) [8]. Based on the various literature and researches the threshold values for perceptible and acceptable color changes in the CIELAB system was proven to be ΔE*ab > 1.2 and ΔE*ab ≤ 2.7, respectively. Also, there are another reliable method to measure color differences through comparison with 50:50% perceptibility and acceptability visual thresholds [8, 9]. 

Artificial aging methods, particularly thermomechanical cycling, are widely used to simulate intraoral thermal and mechanical stresses that can accelerate material degradation. Such aging has been shown to alter surface and mechanical properties of CAD/CAM restorative materials, including changes in surface characteristics relevant to wear and roughness. Therefore, evaluating surface roughness and stainability before and after thermomechanical aging provides a more clinically meaningful estimate of performance than baseline testing alone [10]. 

Accordingly, this study aimed to evaluate how fabrication technique (3D printing versus milling) influences the surface roughness and stainability of a newly introduced resin occlusal veneer material following thermomechanical aging. The null hypothesis was that the fabrication technique would have no significant effect on either surface roughness or color stability after aging.

## Materials and methods

### Study design

This in-vitro study investigated the effect of fabrication technique (subtractive milling versus additive 3D printing) on surface roughness (Ra) and color change (ΔE) of resin occlusal veneers before and after thermomechanical aging. The study was conducted on a standardized prepared typodont mandibular molar and epoxy resin replica; therefore, no human participants were involved and all the steps were done in fixed department at faculty of dentistry Cairo university.

### Materials

All materials, manufacturers, compositions, and lot numbers are reported in supplementary Table 1 (NanoKsa G-Plus disc and resin; finishing/polishing system; dual-cure self-adhesive resin cement; coffee staining medium).

### Sample size calculation

G*Power (v3.1.9.7) [11] was employed to calculate the sample size by a two-sided test of the null hypothesis that there is no difference would be found between different tested groups regarding surface roughness where (α = 0.05, power = 80%) assuming an effect size (d = 1.695) for surface roughness based on previously reported data [12]. The required sample size was 14 specimens (7/group). This was increased by 25% to compensate for possible procedural errors, resulting in 18 specimens (*n* = 9/group).

### Master die preparation

**Fig. 1:**
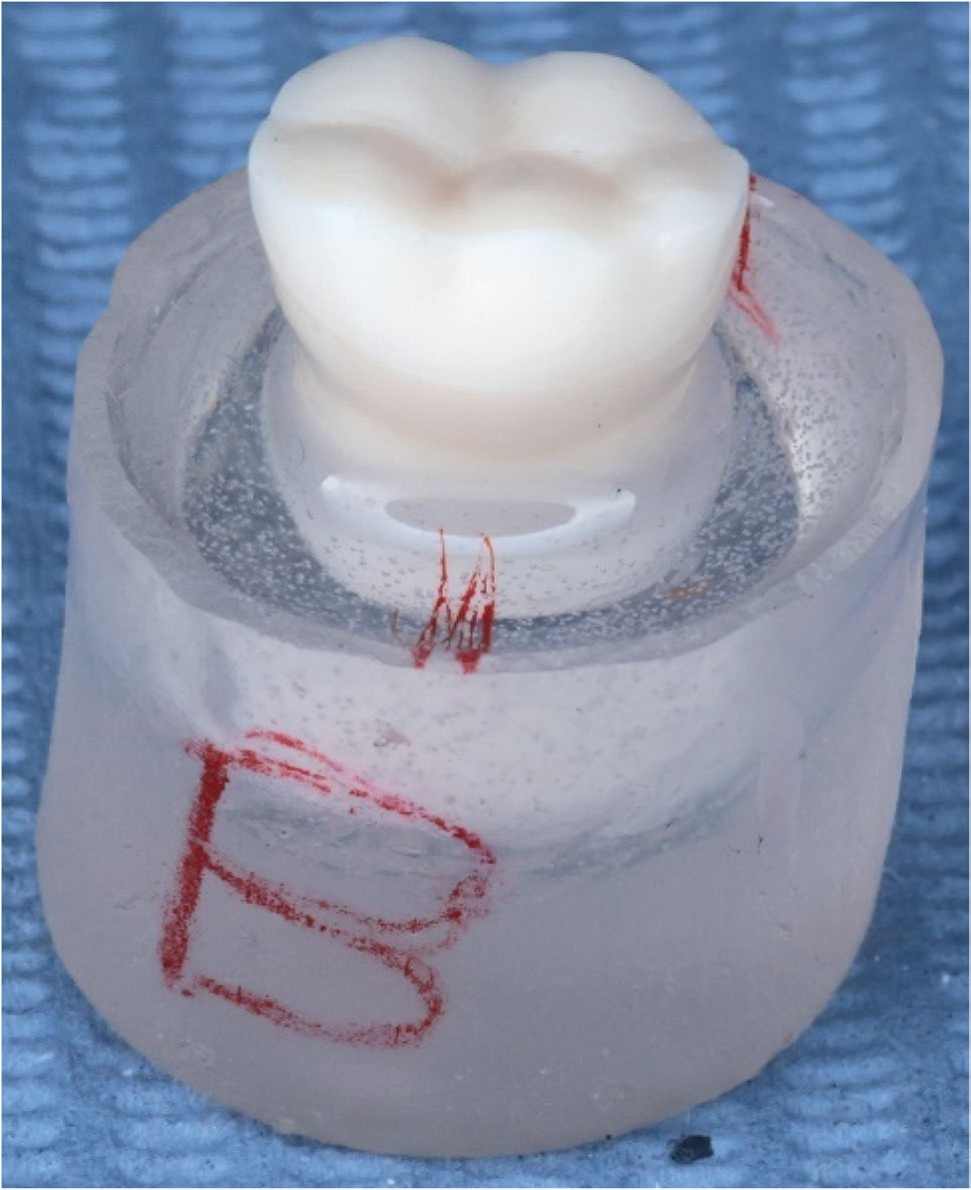
Prepared typodont on egypoxy base (Buccal view)

A typodont mandibular molar was prepared to receive a planar occlusal veneer using a high-speed handpiece and tapered diamond bur mounted to a custom paralleling device to standardize angulation. Occlusal reduction was standardized to 1.0 mm at fissures and 1.5 mm at cusps, with rounding of sharp angles and smoothing of the preparation according to manufacturer’s instructions [6]. Reduction was verified using a silicone putty index (Zhermack zeta plus, Zhermack SPA, Italy) (bucco-lingual cut) to ensure uniform clearance. The prepared typodont was embedded in epoxy resin (Egypoxy, Egypt) within a PVC tube, positioned 2 mm apical to the CEJ, and allowed to set completely which is denoted in Fig. 1.

### Fabrication of epoxy resin dies

Eighteen epoxy replica dies were fabricated from the master die using a silicone putty (Zhermack zeta plus, Zhermack SPA, Italy) mold within a plastic ring. After the impression set according to the manufacturer’s instructions, epoxy resin was mixed (A: B ratio 3:1 by weight), vacuum-mixed for 3 min at low speed, poured into the mold, and allowed to set for 24 h to obtain standardized dies.

### Group allocation

Epoxy dies were restored with full-anatomic occlusal veneers and allocated into two groups (*n* = 9 each) in accordance with fabrication technique: Group M (Milled CAD/CAM): occlusal veneers fabricated from (NanoKsa G-Plus disc, USA) and Group P (3D-printed): occlusal veneers fabricated from (NanoKsa G-Plus resin, USA). Specimens were numbered sequentially to track measurements and analyses.

### Digital acquisition and design

**Fig. 2:**
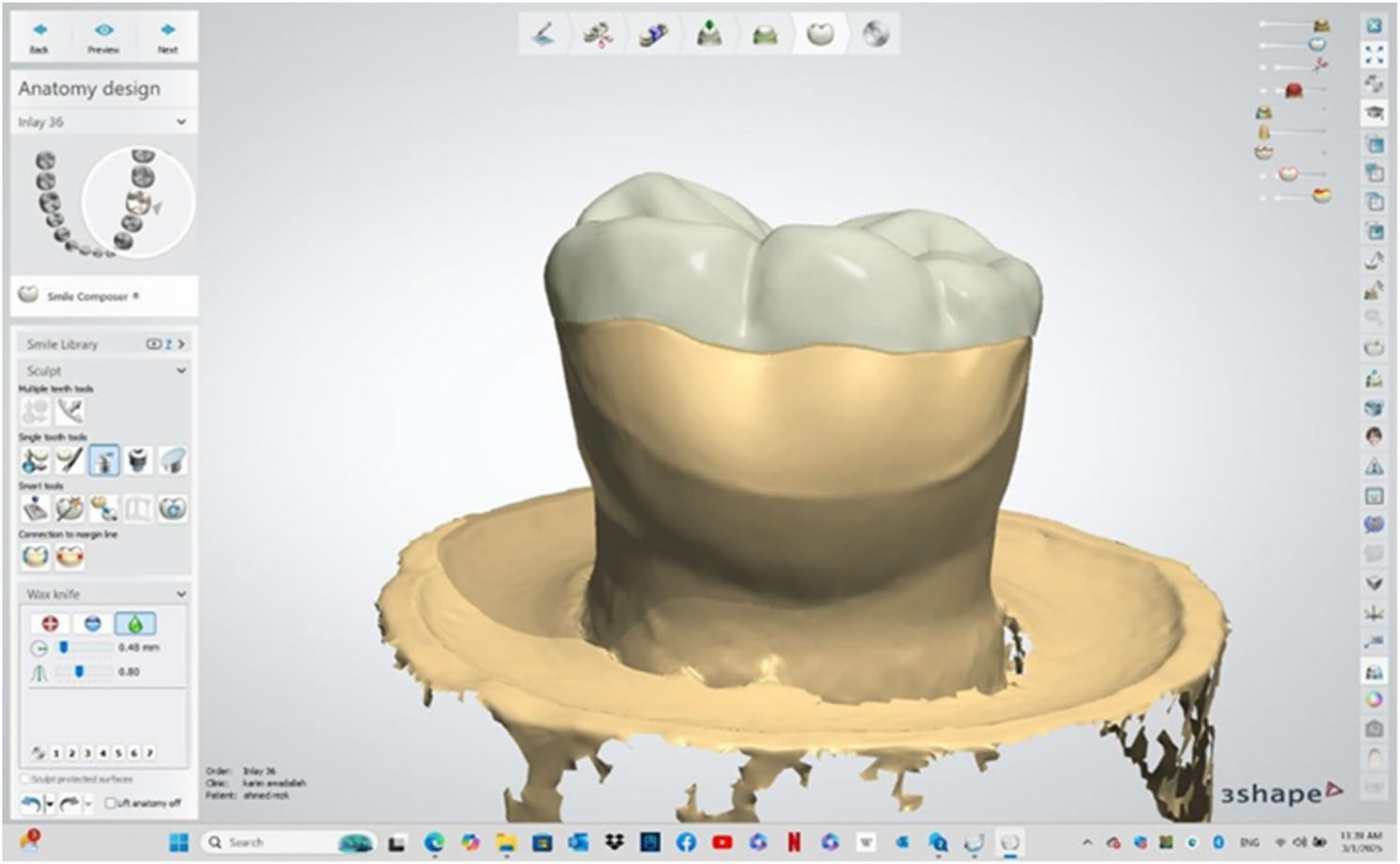
virtual occlusal veneer showing estimated axial thickness on CAD software (buccal view)

The master die was digitized using a laboratory scanner (3Shape E4, Denmark). Occlusal veneers were designed using CAD software with standardized parameters for all specimens: cement space 50 μm, occlusal thickness 1.5 mm on cusps, and 1.0 mm in fissures according to manufacturer instructions [6]. External contours were kept identical across groups, and the same STL file was used for both fabrication methods to ensure geometric standardization which is denoted by Fig. 2.

### Fabrication protocols

*CAD/CAM milling (Group M)*: The STL file was exported to a 5-axis milling unit (REDON HYBRID, Turkey) and veneers (n = 9) were milled from a NanoKsa G-Plus disc (Ø 98 mm). Seating and adaptation were verified on the corresponding epoxy dies.

*3D printing (Group P)*: The same STL file was exported to a DLP printer (INOX S2 Series, USA) and printed using the manufacturer-recommended settings. Key parameters included 405-nm wavelength, layer thickness 50 μm and standardized build orientation with the occlusal surface facing the build platform at a 2-mm offset. After printing, specimens were cleaned in 99% isopropyl alcohol in an ultrasonic bath for 5 min, air-dried, and post-cured according to the manufacturer’s protocol (specified flash cycles from both intaglio and occlusal sides). Final seating was verified on the corresponding epoxy dies.

### Finishing and polishing

All specimens underwent a standardized finishing and polishing protocol using the same sequence and instruments for both groups (Kenda kit (Kenda AG, Switzerland), Sof-Lex discs (3 M/ESPE, St paul, USA), polishing brush, and aluminum-oxide polishing paste (cosmedent, USA) according to manufacturers’ instructions. For standardization and operation calibration protocol including rotational speed ranged from 10,000 to 20,000 rpm for abrasive discs and a maximum of 5,000 rpm for polishing pastes to avoid surface overheating with low pressure applied in a uni-direction starting from heavy removal using bigger disc (approx. 40 μm) till surface gloss using paste. Specimens with evident defects or incomplete seating were excluded and remade prior to testing.

### Cementation protocol

All veneers were cemented to their corresponding epoxy dies using dual-cure self-adhesive resin cement (RelyX U200,3 M/ESPE, USA). Cement was mixed per manufacturer instructions and applied to the intaglio surface. Veneers were seated with finger pressure, then a custom cementation device was used to apply a constant axial load of 49 N (≈ 5 kg) during initial setting to standardize seating. Excess cement was removed after brief tack curing, followed by final light curing from multiple aspects (occlusal, buccal, lingual, mesial, distal) using a curing unit delivering 1000–1200 mW/cm² for 20 s per surface.

### Thermomechanical aging

All specimens underwent thermomechanical aging (Robota automated thermal cycler; BILGE, Turkey) using a computer-controlled chewing simulator integrated with thermocycling. In terms of thermocycling, a5000 cycles was performed between 5 °C and 55 °C with a dwell time of 25 s and transfer time of 10 s. Chewing simulation was performed for 75,000 cycles at 49 N using a 2.5-mm diameter steel antagonist under standardized vertical and horizontal movements (vertical: 3 mm; horizontal: 1 mm; frequency: 1.6 Hz) in deionized water with simultaneous thermocycling. Selection of these parameters was approximately equated to 6 months of functional clinical service [13]. 

### Color measurements and stainability assessment

#### Baseline color measurement

Color was measured using a calibrated digital spectrophotometer (Vita Easyshade Advance 4.0, Germany). Measurements were performed by positioning the probe tip perpendicular (90°) at the center of each specimen. CIE L*a*b* values were recorded in an Excel sheet. To ensure standardization the whole workflow for stainability and color stability was done by same operator.

### Coffee staining protocol

A coffee solution was prepared by dissolving 3.6 g of instant coffee (Nescafe, Switzerland) in 300 mL boiling distilled water with stirring for 10 min, followed by filtration. Specimens were immersed for 7 days at room temperature. The solution was renewed regularly to minimize fermentation and deposition. Before post-staining measurements, specimens were gently cleaned using a soft brush for 1 min and rinsed.

### Calculation of color difference (ΔE)

Measurements were performed three times for each specimen: at baseline, after 7-day coffee immersion and after thermomechanical aging then the mean Lab values (ΔE) were calculated.

Calculation of color change (ΔE) was acquired using: ΔE = [(ΔL)² + (Δa)² + (Δb)²]¹ᐟ², where ΔL, Δa, and Δb represent the differences in L*, a*, and b* values between time points [14]. 

### Surface roughness (Ra) assessment

**Fig. 3:**
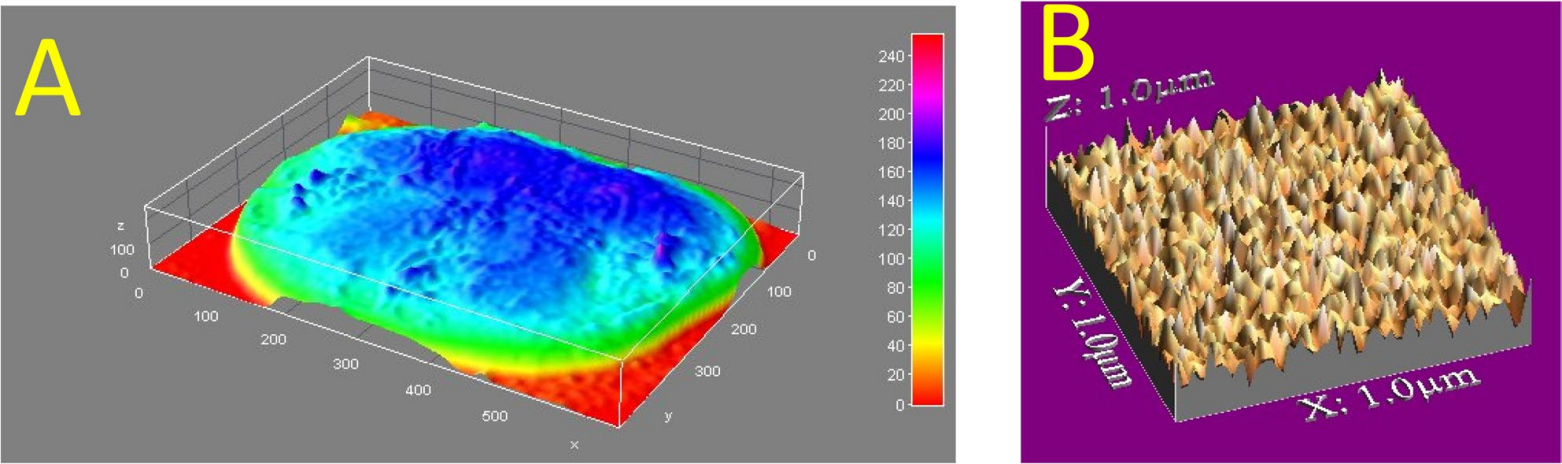
showing (**A**) Surface plot diagram, (**B**) 3D image tomography

Surface roughness was evaluated using a non-contact optical profilometry (Guangdong, China) before and after thermomechanical aging. WSxM software (Version 5.0 Develop 4.1 manufacturered by Nanotec Electrónica S.L.**)** was used to calculate average of heights (Ra) and maximum heights expressed in μm, which can be assumed as a reliable index of surface roughness and expressed in surface plot and 3D tomography which are denoted by Fig 3. Standardized imaging parameters were applied for all specimens. For each specimen, three scans were obtained (central and two peripheral areas) over a standardized scan area

### Statistical analysis

Statistical analyses were performed using SPSS (version 26). Data normality was assessed using Shapiro–Wilk and Kolmogorov–Smirnov tests. For intergroup comparisons (milled vs. printed) at each time point, an independent samples t-test was used. For intragroup comparisons across time points (baseline vs. post-staining vs. post-aging), a repeated-measures ANOVA (or paired t-tests with correction was performed followed by Tukey’s post hoc test for multiple comparisons. The significance level was set at *p* ≤ 0.05.

## Results

Surface roughness (Ra) and color change (ΔE) are summarized in Tables 1 and 2. At baseline, the 3D-printed occlusal veneers demonstrated significantly higher surface roughness than the CAD/CAM-milled group (Mean = 0.27 while SD = 0.007 vs. Mean = 0.24 while SD = 0.02; *p* = 0.05 respectively). After thermomechanical aging, no statistically significant difference was evident in surface roughness between the two groups (Mean = 0.26 while SD = 0.021 vs. Mean = 0.27 while SD = 0.016; *p* = 0.50). Intragroup comparisons showed no significant change in Ra after aging in either the milled group (*p* = 0.09) or the 3D-printed group (*p* = 0.45) (Table 1).

**Table 1:** Surface roughness (Ra) of milled vs. 3D-printed occlusal veneers before and after thermomechanical aging

Outcome (Ra)	Milled (*n* = 9) Mean (SD)	3D printing (*n* = 9) Mean (SD)	Intergroup *p*-value
Baseline (before aging)	0.24 ( 0.02)	0.27 ( 0.007)	0.05*
Post-aging (after aging)	0.27 ( 0.016)	0.26 ( 0.021)	0.50
Intragroup *P*-value	0.09	0.45	

*P-value is significant at 0.05 level or less

Regarding stainability, the printed group samples exhibited significantly greater color change than the CAD/CAM-milled group across all evaluated intervals (Table 2). Color change from baseline to after coffee staining (1 week) was significantly higher in the 3D-printed group (Mean = 34.33, SD = 6.14) compared with the milled group (Mean = 13.14 while SD = 2.31; *p* = 0.001). Following thermomechanical aging, the additional color change from post-staining to post-aging remained significantly greater in the 3D-printed group (Mean = 18.78 while SD = 3.85) than in the milled group (Mean = 2.66 while SD = 1.89; *p* < 0.001). Overall color change from baseline to post-aging was also markedly higher in 3D-printed veneers (Mean = 40.30 while SD = 5.78) than in CAD/CAM-milled veneers (Mean = 12.53 while SD = 3.19; *p* < 0.001). Intragroup analysis revealed statistically significant differences in ΔE across the assessment intervals in both groups (CAD/CAM: *p* < 0.001; 3D printing: *p* = 0.004).

**Table 2:** Color change (ΔE) of milled vs. 3D-printed occlusal veneers across testing interval

ΔE interval	Milled (*n* = 9) Mean (SD)	3D printing (*n* = 9) Mean (SD)	Intergroup *p*-value
Baseline → after coffee staining (1 week)	13.14 (2.31)	34.33 (6.14)	0.001*
After staining → after thermomechanical aging	2.66 (1.89)	18.78 (3.85)	0.000*
Baseline → after thermomechanical aging	12.53 (3.19)	40.30 (5.78)	0.000*
Intragroup *P*-value	< 0.001*	0.004*	

*P-value is significant at 0.05 level or less

## Discussion

In this study, we looked at the effect of fabrication technique (CAD/CAM milling versus 3D printing) on the surface roughness and color stability of INOX resin occlusal veneers before and after thermomechanical aging. Overall, we found that 3D-printed veneers exhibited significantly higher baseline surface roughness than milled veneers; however, this difference was no longer statistically significant after aging. In contrast, color change (ΔE) was markedly higher in the 3D-printed group across all evaluation intervals, which suggests that stainability and optical stability were more sensitive to fabrication technique than surface roughness.

The INOX resin used in this study is a recently introduced high-performance polymer. While its mechanical properties have been preliminarily explored (Abdelrahim et al., 2025), evidence regarding how different digital fabrication workflows influence its surface and optical performance, particularly after aging, remains scarce [15, 16]. 

In our study we used epoxy resin dies instead of natural teeth for many reasons first of all it was difficult to obtain 18 samples with same occlusal anatomy and configuration as extracted natural teeth by the time undergo shrinkage, second, in order to decrease any variables and be sure that all samples are exact replica and identical to the master die, third, epoxy dies characterized by its higher dimensional accuracy and it’s elastic modulus comparable to the natural dentin (12.9 GPa) [17]. 

In this study, baseline roughness values were higher in the 3D-printed group. This could be explained by the layer-by-layer nature of additive manufacturing, which may generate surface micro-irregularities or interlayer features that are difficult to completely eliminate by finishing and polishing, especially on anatomically complex occlusal surfaces. After thermomechanical aging, roughness values converged between groups and intragroup differences were not statistically significant. thereby reducing initial differences between fabrication techniques [8, 9].

After aging, the difference between the two groups becomes less pronounced. This starts by inducing unbalanced degradation, where the initially smoother surface of milled materials undergoes a distinct deterioration opposed to the earlier rougher and less stable surface of 3D-printed materials. 3D-printed materials often have higher initial roughness due to layer over layer construction (Like stair in shape) where elevated stress beside cyclical environment forces both materials toward a similar, higher-roughness state, that’s why minimizing initial remarkable gap values between the two fabrication techniques [10]. 

A clear difference in stainability was observed between the two groups represents a key finding of this study. The 3D-printed group revealed a remarkably higher ΔE values after coffee staining and maintained greater discoloration after subsequent thermomechanical aging. the recorded ΔE values (reaching up to 40.30) were markedly higher than the commonly accepted clinical threshold (~ 2.7), representing a marked and clinically unacceptable level of discoloration rather than a minor perceptible difference. This higher ΔE mean difference values observed in this study may be attributed due to immersion of the two groups in coffee solution which was mentioned by many studies that using strong and high concentration of beverage solution like coffee for long immersion time can cause a marked increase in values of ΔE which can raise up to ≥ 12 ( which in turn necessitate restoration replacement ) and also may be due to during the fabrication process of 3D printed specimens which may be influenced by the addition of layer by layer and the post curing step which may also play role in this elevation of ΔE values. These results should be interpreted with caution because the staining workflow represents increased in vitro conditions that may exaggerate discoloration compared to clinical wise [18, 19]. 

Discoloration of resin-based materials has been associated with water sorption, polymer matrix degradation, and the presence of unreacted residual monomers, all of which may be influenced by fabrication technique and post-processing efficiency [16, 20]. The higher discoloration observed in printed specimens may therefore be related to polymer network characteristics and interlayer interfaces that facilitate pigment penetration. Additionally, the diverse equipment used for the post-curing process and the variation in the time limit of the post-curing for each type of additively manufactured material may be the reason for these results. Where superior post-curing productivity defined by remarkable raise in the degree of conversion (DC) and minimized residual monomers which in turn notably reduces stainability, water sorption, and causing discoloration over time and vice versa in case of low post curing time. This interpretation is literature -based that should be clarified and undergoing further researches as plausive rather than conclusive [9, 21]. 

Milled resin Inox are fabricated from pre-polymerized blanks processed using high-pressure. This yields more compact, distinct arranged structure linked with high density of polymer chains and minimal residual monomer. However, 3D printed one is constructed using layer-by-layer photo-polymerization. Which in turn leads to lower degrees of conversion (nearly 65–75%) and a lower-density mesh that demand post-curing to enhance mechanical properties. This polymer network variation between the 2 groups have influence on color stability where the milled group poses greater color stability and minimum discoloration rate attributable to the higher cross-link density, which hinder any of staining agents from entering the material on contrary the 3D printed veneers has distinct discoloration rate due to their high permeability and lower conversion rate make them more vulnerable to more staining and discoloration from water sorption and chromogenic beverage solutions like coffee and tea. These structural differences may explain the increased pigment penetration and water sorption observed in the 3D-printed group, ultimately contributing to the markedly higher ΔE values [22]. 

Coffee was selected as a staining medium because of its high staining potential and its ability to cause both surface adsorption and deeper absorption of chromogens within resin matrices [20].

ΔE _ab_ (CIE LAB) was eligible to be used in this study thanks to its simplicity and consistency beside it provides a straightforward and clear formula which is commonly mentioned in many previous as the most widespread mean in measuring stainability of different materials like resin-based materials [14]. 

Our ongoing research is consistent with previous comparative studies. Bozoğulları and Temizci reported increased roughness and color changes in resin-based CAD/CAM materials after thermocycling, with 3D-printed materials showing greater susceptibility to discoloration than milled counterparts [9]. Similarly, Morsy et al. demonstrated that fabrication technique significantly affects surface behavior and wear characteristics of printed and milled occlusal veneers following thermomechanical aging, supporting the concept that additive manufacturing may be more sensitive to aging-related optical changes [12]. Furthermore, a recent systematic review and meta-analysis by Alghauli MA et al. who concluded that while 3D-printed restorations may exhibit remarkable workflow advantages on the contrary, their optical manner and aging reaction relies on material and operational factors when compared with milled ones. they also ended with the conclusion that using 3D printing polymers in clinical dentistry are not accurately assessed concerning their visual and transparency characteristics. Eventually, future investigations on the effect of testing various printing conditions, such as layer positioning and thickness and effect of post- curing stage on the color and optical behavior of 3D printing polymers should asset in enhancing the final esthetic outcome of these materials [5].

From a clinical perspective, surface roughness values obtained after aging remained within the low micrometer range and did not differ significantly between fabrication techniques, suggesting limited clinical impact regarding plaque retention when standardized finishing and polishing are applied. In contrast, the magnitude of color change observed in the 3D-printed group exceeded commonly reported acceptability thresholds for dental color differences, indicating that discoloration may represent the primary clinical limitation for printed occlusal veneers under staining and aging conditions [23]. Therefore, fabrication technique should be carefully considered when esthetic longevity is a priority.

Methodological standardization, including uniform CAD design parameters and controlled thermomechanical aging, was implemented to minimize confounding variables and improve comparability between groups [10, 24]. The use of non-contact optical profilometry allowed three-dimensional surface assessment without the limitations of stylus contact on anatomically contoured surfaces, enhancing the reliability of roughness measurements [25]. Instrumental color assessment using a spectrophotometer further reduced subjectivity and enabled quantitative evaluation of color changes beyond visual perception limits [23].

Despite these strengths, the present study has limitations. As an in-vitro investigation, it cannot fully replicate the complex oral environment. In addition, only single shade and staining agent of material was used which in turn may affect in one way or another the color stability which can vary between shades of the same resin and eventually affecting the generalization findings of stainability outcome. and factors such as toothbrushing abrasion, pH cycling, and salivary effects were not included. In addition, only one 3D printing system and set of processing parameters were evaluated; therefore, the results should not be generalized to all additive manufacturing protocols. Future studies should investigate the impact of different post-curing times and temperatures, as well as the application of surface sealants, on the color stability of 3D-printed resins., as well as validate these findings through clinical trials.

## Conclusion

Given the limitations of this in-vitro study, fabrication technique influenced the optical performance of resin occlusal veneers more than their surface roughness. Although baseline surface roughness was higher in the 3D-printed group, thermomechanical aging resulted in comparable Ra values between milled and printed veneers. In contrast, 3D-printed veneers exhibited significantly greater color change (ΔE) after coffee staining and after thermomechanical aging compared with milled veneers exceedingly the commonly reported clinical acceptability thresholds, which suggests lower color stability under the tested conditions which may limit its clinical use in esthetically demanding situations.

### Recommendations

Prefer milled veneers when esthetics are critical; use 3D-printed with caution due to higher discoloration risk, optimize printing/post-curing parameters and evaluate surface coatings/sealants to improve color stability and Future studies should include toothbrushing/pH cycling, multiple staining agents, and clinical trials to confirm in-vitro findings.

## Supplementary Information

Supplementary Material 1.

## Data Availability

The datasets generated and/or analyzed during the current study are available from the corresponding author on reasonable request.

## Abbreviations

CAD/CAM: Computer-Aided Design/Computer-Aided Manufacturing
DLP: Digital Light Processing
Ra: Arithmetic mean surface roughness
ΔE: Color difference (CIE L*a*b*)
CIE: Commission Internationale de l’Eclairage
L*: Lightness coordinate in CIE L*a*b* system
a*: Red–green axis coordinate in CIE L*a*b* system
b*: Yellow–blue axis coordinate in CIE L*a*b* system
IPA: Isopropyl alcohol
HPP: High-performance polymers
PEEK: Polyetheretherketone
CEJ: Cementoenamel junction
STL: Standard Tessellation Language (3D model file format)
SPSS: Statistical Package for the Social Sciences
