# Supplementary material for: Effect of fabrication technique on surface roughness and color stability of novel resin occlusal veneers after thermomechanical aging: an in-vitro study
Source: BMC Oral Health. 2026 May 18;26:931. doi: 10.1186/s12903-026-08536-8 (PMC13227696; doi:10.1186/s12903-026-08536-8)
Supplement: Supplementary file 1 — Supplementary Material 1. [file 12903_2026_8536_MOESM1_ESM.docx]

**Table S1. Materials used in the study (manufacturer-reported composition and lot numbers**

| **Category / Use** | **Material (trade name)** | **Manufacturer (Country)** | **Manufacturer-reported composition** | **Lot No.** |
| --- | --- | --- | --- | --- |
| CAD/CAM subtractive material (milling) | **NanoKsa G-Plus Disc** | INOX MENA Co. (USA) | High-performance polymers with nano-zirconia and carbon | **4293** |
| Additive material (3D printing) | **NanoKsa G-Plus resin** | INOX MENA Co. (USA) | Resin filled with high-performance polymers (HPP), nano-zirconia and carbon | **4293** |
| Finishing & polishing system | **Sof-Lex Finishing & Polishing System** | 3M/ESPE, St Paul, MN (USA) | Urethane-coated abrasive discs containing aluminum oxide grits (coarse to superfine; color-coded) | **7982** |
| Luting agent (cementation) | **RelyX U200** (dual-cure, self-adhesive resin cement) | 3M ESPE Dental Products (USA) | Methacrylate monomers containing phosphoric acid groups, methacrylate monomers, silanated fillers, initiator components, stabilizers, rheologic additives, alkaline fillers, pigments | **714236** |
| Staining medium | **Nescafé Classic** | Nestlé (Switzerland) | Instant coffee (roasted coffee beans, spray-dried) | **6221007** |
